# Supplementary material for: A randomised Trial of Autologous Blood products, leukocyte and platelet-rich fibrin (L-PRF), to promote ulcer healing in LEprosy: The TABLE trial
Source: PLoS Negl Trop Dis. 2024 May 2;18(5):e0012088. doi: 10.1371/journal.pntd.0012088 (PMC11093377; doi:10.1371/journal.pntd.0012088)
Supplement: S1 Table — (DOCX) [file pntd.0012088.s001.docx]

**S1 Table.** Consort Flow Diagram

**Not randomised (n=2)**

Not leprosy patient=1

During baseline data collection clinical team observed and decided to close the wound by suturing=1

**Assessed for eligibility** **(n=283)**

**Excluded (n=151)**

Not meeting inclusion or meeting exclusion criteria (n=141)

Declined to participate (n=10)

Enrolment

**Consented (n=132)**

**Randomised (n=130)**

Allocation

Lost to follow-up (n=0)

Withdrawn (n=1)

Deaths (n=0)

*See table 7 for more details*

Analysed

Analysed

Follow-Up at 42 days

Follow-Up at 6 months

**Dressing changes with normal saline (n=65)**

*See table 7 for more details*

**Dressing changes with L-PRF matrix (n=65)**

*See table 7 for more details*

**Analysed at 42 days (n=65)**

Note: *All participants were included and censored if needed at 42 days or their withdrawal date as appropriate.*

**Analysed at 42 days (n=65)**

Note: *All participants were included and censored if needed at 42 days or their withdrawal date as appropriate.*

Lost to follow-up (n=0)

Withdrawn (n=0)

Deaths (n=0)

*See table 7 for more details*

Lost to follow-up (n=0)

Withdrawn (n=1)

Deaths (n=0)

*See table 7 for more details*

**Analysed at 70 days (n=65)**

Note: *All participants were included and censored if needed at 70 days or their withdrawal date as appropriate.*

*See table 7 for more details*

**Analysed at 70 days (n=65)**

Note: *All participants were included and censored if needed at 70 days or their withdrawal date as appropriate.*

*See table 7 for more details*

Lost to follow-up (n=1)

Withdrawn (n=0)

Deaths (n=0)

*See table 7 for more details*

**Analysed at 6 months follow-up (n=64)**

Excluded from analysis (n=5)

**Note:** *Excluded because their assessment was outside the window of 6 months ±1 month from randomisation. A sensitivity analysis including all participants was conducted.*

*See table 7 for more details*

Lost to follow-up (n=4)

Withdrawn (n=0)

Deaths (n=0)

*See table 7 for more details*

Lost to follow-up (n=0)

Withdrawn (n=2)

Deaths (n=0)

*See table 7 for more details*

Follow-Up at 70 days

Analysed

**Analysed at 6 months follow-up (n=61)**

Excluded from analysis (n=0)
