# Supplementary material for: A randomised Trial of Autologous Blood products, leukocyte and platelet-rich fibrin (L-PRF), to promote ulcer healing in LEprosy: The TABLE trial
Source: PLoS Negl Trop Dis. 2024 May 2;18(5):e0012088. doi: 10.1371/journal.pntd.0012088 (PMC11093377; doi:10.1371/journal.pntd.0012088)
Supplement: S2 Table — (DOCX) [file pntd.0012088.s002.docx]

**S2 Table.** Reasons for participant exclusion

| **Reasons^1^** | **Number of participants excluded**  **(N=151)** |
| --- | --- |
| **Did not meet inclusion criteria** | **101 (66.9%)** |
| Ulcer surface area<2 cm^2^ or ulcer surface area >20 cm^2^ | 76 (75.2%) ^2^ |
| Ulcer is not clean, dry and free from clinical infection | 18 (18.0%) |
| Patient didn’t have a chronic foot ulcer | 9 (8.9%) |
| Patient < 18 years old | 4 (4.0%) |
| Patient is not able to understand and willing to voluntarily sign an informed consent document prior to any study related assessments being conducted | 4 (4.0%) |
| **Meeting exclusion criteria** | **128 (84.8%)** |
| Ulcer surface area<2 cm^2^ or ulcer surface area >20 cm^2^ | 75 (58.6%) ^2^ |
| Wound that has clinical microbial infections | 40 (31.3%) |
| Significant medical condition, laboratory abnormality or psychiatric illness | 19 (14.8%) |
| Diabetes or a diabetic ulcer | 13 (10.2%) |
| Condition that confounds the ability to interpret data from the study (i.e., HIV, chronic Hep B, chronic Hep C or TB Patients  under active treatment) | 6 (4.7%) |
| Haemoglobin count less than 9 gm/dL | 5 (3.9%) |
| Patient requires a skin graft | 3 (2.3%) |
| Erythema Nodosum Leprosum (ENL) or a leprosy reaction under steroid treatment | 3 (2.3%) |
| High blood pressure ( >150 systolic mmHg) | 2 (1.6%) |
| Patient is pregnant or a nursing female | 2 (1.6%) |
| Platelet count less than 100x10^3^/ul | 0 (0%) |
| Patient returned to the hospital, having already taken part in the trial | 0 (0%) |
| **Did not want to participate in trial** | **10 (6.6%)** |

*1: Reasons are not mutually exclusive so may total to greater than 100%.*

*2: There were 7 participants for whom the area of their ulcer was categorised as being both between 2 cm^2^ and 20 cm^2^ and outside these limits.*
