# Supplementary material for: A randomised Trial of Autologous Blood products, leukocyte and platelet-rich fibrin (L-PRF), to promote ulcer healing in LEprosy: The TABLE trial
Source: PLoS Negl Trop Dis. 2024 May 2;18(5):e0012088. doi: 10.1371/journal.pntd.0012088 (PMC11093377; doi:10.1371/journal.pntd.0012088)
Supplement: S3 Table — (DOCX) [file pntd.0012088.s003.docx]

**S3 Table.** Description of the intervention (up to 70 days)

|  | **Dressing changes with normal saline ^1^ (N=65)** | **Dressing changes with**  **L-PRF matrix (N=65)** | **Total (N=130)** |  |
| --- | --- | --- | --- | --- |
| **Total time taken for dressing change (min) ^2^** | | | |  |
| Number of participants | 65 | 65 | 130 |  |
| Total number of dressing changes | 895 | 780 | 1675 |  |
| Mean (SD) | 9.9 (4.7) | 23.9 (6.6) | 16.4 (9.0) |  |
| Min - Max | 3.0 – 39.0 | 3.0 – 62.0 | 3.0 – 62.0 |  |
| Median | 9.0 | 25.0 | 12.0 |  |
| P_25_ - P_75_ | 8.0 – 10.0 | 22.0 – 28.0 | 8.0 – 25.0 |  |
| Number of dressing changes in which the total time for dressing change was not reported | 0 | 1 | 1 |  |
| **Dressing changes with L-PRF matrix** | | | | |
| Number of participants who had at least one dressing changes with L-PRF matrix | 7^3^ | 65 | 72 |  |
| Number of dressing changes with L-PRF matrix | 43 | 720 | 763 |  |
| Number of dressing changes in which whether an L-PRF matrix was performed or not, was not reported | 0 | 0 | 0 |  |
| **Volume of blood drawn (in ml)** | |  |  |  |
| Number of participants for whom the  volume of blood drawn was reported | 7^3^ | 65 | 72 |  |
| Number of dressing changes in which the volume of blood drawn was reported | 43 | 720 | 763 |  |
| Mean (SD) | 14.2 (7.0) | 13.9 (7.3) | 13.9 (7.3) |  |
| Min - Max | 10.0 – 30.0 | 10.0 – 70.0 | 10.0 – 70.0 |  |
| Median | 10.0 | 10.0 | 10.0 |  |
| P_25_ - P_75_ | 10.0 – 20.0 | 10.0 – 20.0 | 10.0 - 20.0 |  |
| Number of dressing changes in which the volume of blood drawn was not reported | NA | 61 | NA |  |
| **Number of L-PRF matrices applied on ulcer** | | | |  |
| Number of participants who had at least one dressing change in which the  number of L-PRF matrices applied on  ulcer was reported | 7^3^ | 65 | 72 |  |
| Number of dressing changes in which  the number of L-PRF matrices applied  on ulcer was reported | 43 | 720 | 763 |  |
| Mean (SD) | 1.4 (0.7) | 1.4 (0.7) | 1.4 (0.7) |  |
| Min - Max | 1.0 – 3.0 | 1.0 – 7.0 | 1.0 – 7.0 |  |
| Median | 1.0 | 1.0 | 1.0 |  |
| P_25_ - P_75_ | 1.0 – 2.0 | 1.0 – 2.0 | 1.0 – 2.0 |  |
| Number of dressing changes in which the number of L-PRF matrices applied on ulcer  was not reported | NA | 61 | NA |  |

*1: If a participant in the dressing changes with normal saline group had an unhealed ulcer at 42 days and the responsible clinician felt that progress was slow and the participant wished, then the participant could transfer to receive the intervention.*

*2: Figures on the total time taken for dressing change were calculated across all dressing changes and all participants for whom the total time taken for dressing change was reported.*

*3: these 7 participants crossed over from the control to intervention group after 42 days as allowed by protocol. There were no cross overs from intervention to control.*
