# Supplementary material for: A randomised Trial of Autologous Blood products, leukocyte and platelet-rich fibrin (L-PRF), to promote ulcer healing in LEprosy: The TABLE trial
Source: PLoS Negl Trop Dis. 2024 May 2;18(5):e0012088. doi: 10.1371/journal.pntd.0012088 (PMC11093377; doi:10.1371/journal.pntd.0012088)
Supplement: S4 Table — (DOCX) [file pntd.0012088.s004.docx]

**S4 Table.** Protocol deviations by group

| **Deviation description** | **Dressing changes with normal saline (N=65)** | **Dressing changes with L-PRF matrix (N=65)** |
| --- | --- | --- |
| Participants were randomised before the baseline assessment. | 1 (2%) | 3 (5%) |
| 6-month follow-up assessment was after the time window. | 2 (3%)^1^ | 0 (0%) |
| Participant did not receive the allocated intervention (L-PRF) at the 1^st^ and 2^nd^ dressing change assessment because they had fever. | 0 (0%) | 1 (2%) |
| Participant was given alternate day dressing change in three dressing changes, due to moderate growth of Pseudomonas aeruginosa isolated from trial ulcer. | 1 (2%) | 0 (0%) |
| Participant withdrew from the trial and went home without informing the trial team and thus therefore haemoglobin and platelet data were not collected at discharge. | 0 (0%) | 1 (2%) ^2^ |
| Participant did not receive the allocated intervention (L-PRF) at the 13^th^ and 14^th^ dressing change assessment because they had COVID-19. | 0 (0%) | 1 (2%) |

*1: For another 3 participants, their 6-month follow-up assessments were after the time window, but a general file note was created and therefore, a protocol deviation form was not completed for these cases.*

*2: For another participant, haemoglobin and platelet data were not collected at discharge because they withdrew from the trial and had to leave immediately. The trial team completed a file note.*
