# Supplementary material for: A randomised Trial of Autologous Blood products, leukocyte and platelet-rich fibrin (L-PRF), to promote ulcer healing in LEprosy: The TABLE trial
Source: PLoS Negl Trop Dis. 2024 May 2;18(5):e0012088. doi: 10.1371/journal.pntd.0012088 (PMC11093377; doi:10.1371/journal.pntd.0012088)
Supplement: S5 Table — (DOCX) [file pntd.0012088.s005.docx]

**S5 Table.** Full table of baseline characteristics by group

|  |  | **Dressing changes**  **with normal saline**  **(N=65)** | **Dressing changes**  **with**  **L-PRF matrix**  **(N=65)** | **Total (N=130)** |
| --- | --- | --- | --- | --- |
| **Variables used in covariate adjustment** | |  |  |  |
| Trial ulcer Area ^1^ (cm^2^) -PUSH tool | n | 65 | 64 | 129 |
|  | Mean (SD) | 3.9 (3.0) | 3.7 (2.8) | 3.8 (2.9) |
|  | Min - Max | 0.6 – 12.5 | 0.9 – 11.3 | 0.6 – 12.5 |
|  | Missing | 0 | 1 | 1 |
| Date of birth known? | Yes | 27 (41.5%) | 29 (44.6%) | 56 (43.1%) |
|  | No | 38 (58.5%) | 36 (55.4%) | 74 (56.9%) |
|  | Missing | 0 | 0 | 0 |
| Age at randomisation (years) ^2^ | n | 65 | 65 | 130 |
|  | Mean (SD) | 53.6 (17.1) | 54.5 (14.4) | 54.0 (15.8) |
|  | Min - Max | 20.0 – 89.0 | 22.0 – 84.0 | 20.0 – 89.0 |
| **Participant demographics** |  |  |  |  |
| Gender, n (%) | Male | 54 (83.1%) | 49 (75.4%) | 103 (79.2%) |
|  | Female | 11 (16.9%) | 16 (24.6%) | 27 (20.8%) |
| Highest level of education | Never joined formal school | 25 (38.4%) | 26 (40.0%) | 51 (39.2%) |
|  | Can read and write | 16 (24.6%) | 7 (10.8%) | 23 (17.7%) |
|  | Primary level | 13 (20.0%) | 14 (21.5%) | 27 (20.8%) |
|  | Secondary level | 9 (13.9%) | 16 (24.6%) | 25 (19.2%) |
|  | Higher secondary level | 2 (3.1%) | 2 (3.1%) | 4 (3.1%) |
|  | University level | 0 (0%) | 0 (0%) | 0 (0%) |
| **Clinical information** |  |  |  |  |
| Height in cm | n | 65 | 65 | 130 |
|  | Mean (SD) | 157.6 (7.8) | 157.3 (8.5) | 157.5 (8.1) |
|  | Min - Max | 136.5 – 178.0 | 136.5 – 176.0 | 136.5 – 178.0 |
| Weight in kg | n | 65 | 65 | 130 |
|  | Mean (SD) | 54.5 (8.4) | 56.8 (9.9) | 55.7 (9.2) |
|  | Min - Max | 37.5 – 79.3 | 30.0 – 85.3 | 30.0 – 85.3 |
| BMI | n | 65 | 65 | 130 |
|  | Mean (SD) | 22.0 (3.3) | 23.0 (3.7) | 22.5 (3.5) |
|  | Min - Max | 17.0 – 32.5 | 14.9 – 32.5 | 14.9 – 32.5 |
| Blood Pressure – Systolic (mmHg) | n | 65 | 65 | 130 |
|  | Mean (SD) | 112.8 (9.9) | 114.2 (11.8) | 113.5 (10.9) |
|  | Min - Max | 90.0 – 140.0 | 90.0 – 150.0 | 90.0 – 150.0 |
| Blood Pressure – Diastolic (mmHg) | n | 65 | 65 | 130 |
|  | Mean (SD) | 78.0 (7.1) | 77.2 (8.8) | 77.6 (8.0) |
|  | Min - Max | 60.0 – 90.0 | 50.0 – 100.0 | 50.0 – 100.0 |
| Platelet result x 10^3^/ul | n | 65 | 65 | 130 |
|  | Mean (SD) | 284.1 (91.3) | 283.3 (86.1) | 283.7 (88.4) |
|  | Min - Max | 127.0 – 572.0 | 128.0 – 551.0 | 127.0 – 572.0 |
| Haemoglobin result in gm/dL | n | 65 | 65 | 130 |
|  | Mean (SD) | 13.7 (1.7) | 13.6 (2.1) | 13.6 (1.9) |
|  | Min - Max | 10.0 – 18.1 | 9.7 – 18.8 | 9.7 – 18.8 |
| Fasting blood sugar result in mg/dL | n | 65 | 65 | 130 |
|  | Mean (SD) | 83.0 (11.5) | 85.8 (12.0) | 84.4 (11.8) |
|  | Min - Max | 50.0 – 109.0 | 60.0 – 110.0 | 50.0 – 110.0 |
| **Leprosy details** |  |  |  |  |
| Number of years since leprosy diagnosis | n | 65 | 65 | 130 |
|  | Mean (SD) | 20.8 (15.3) | 18.6 (14.2) | 19.7 (14.7) |
|  | Min - Max | 1.0 – 60.0 | 1.0 – 50.0 | 1.0 – 60.0 |
| Oral antibiotic (multi-drug therapy) treatment for leprosy | Ongoing | 5 (7.7%) | 6 (9.2%) | 11 (8.4%) |
|  | Completed | 60 (92.3%) | 58 (89.2%) | 118 (90.8%) |
|  | Unknown | 0 (0%) | 1 (1.6%) | 1 (0.8%) |
| Diseases other than leprosy ^3^ | Yes | 8 (12.3%) | 12 (18.5%) | 20 (15.4%) |
|  | No | 57 (87.7%) | 53 (81.5%) | 110 (84.6%) |
| **VMT/ST** |  |  |  |  |
| VMT/ST | Normal | 0 (0%) | 0 (0%) | 0 (0%) |
|  | Impaired | 65 (100%) | 65 (100%) | 130 (100%) |
| **Among Impaired** |  |  |  |  |
| Any nerve enlarged in leg | Left | 17 (26.2%) | 23 (35.4%) | 40 (30.8%) |
|  | Right | 21 (32.3%) | 15 (23.1%) | 36 (27.7%) |
|  | Nil | 27 (41.5%) | 27 (41.5%) | 54 (41.5%) |
| Any loss of sensation in foot | Left | 26 (40.0%) | 37 (56.9%) | 63 (48.5%) |
|  | Right | 39 (60.0%) | 28 (43.1%) | 67 (51.5%) |
|  | Nil | 0 (0%) | 0 (0%) | 0 (0%) |
| Any loss of motor function in foot | Left | 14 (21.5%) | 18 (27.7%) | 32 (24.6%) |
|  | Right | 18 (27.7%) | 16 (24.6%) | 34 (26.2%) |
|  | Nil | 33 (50.8%) | 31 (47.7%) | 64 (49.2%) |
| Any deformity in foot | Left | 25 (38.5%) | 30 (46.2%) | 55 (42.3%) |
|  | Right | 36 (55.4%) | 26 (40.0%) | 62 (47.7%) |
|  | Nil | 4 (6.1%) | 9 (13.8%) | 13 (10.0%) |
| **Current Ulcer Information** |  |  |  |  |
| Total number of current ulcers, Left foot | n | 65 | 65 | 130 |
|  | Mean (SD) | 0.46 (0.56) | 0.66 (0.62) | 0.56 (0.60) |
|  | Min - Max | 0.0 – 2.0 | 0.0 – 2.0 | 0.0 – 2.0 |
|  | 0 ulcers | 37 (56.9%) | 27 (41.5%) | 64 (49.2%) |
|  | 1 ulcer | 26 (40.0%) | 33 (50.8%) | 59 (45.4%) |
|  | 2 ulcers | 2 (3.1%) | 5 (7.7%) | 7 (5.4%) |
| Total number of current ulcers, Right foot | n | 65 | 65 | 130 |
|  | Mean (SD) | 0.77 (0.66) | 0.57 (0.59) | 0.67 (0.63) |
|  | Min - Max | 0.0 – 2.0 | 0.0 – 2.0 | 0.0 -2.0 |
|  | 0 ulcers | 23 (35.4%) | 31 (47.7%) | 54 (41.5%) |
|  | 1 ulcer | 34 (52.3%) | 31 (47.7%) | 65 (50.0%) |
|  | 2 ulcers | 8 (12.3%) | 3 (4.6%) | 11 (8.5%) |
| Location of the trial ulcer | Left forefoot | 13 (20.0%) | 20 (30.8%) | 33 (25.4%) |
|  | Left midfoot | 11 (16.9%) | 7 (10.8%) | 18 (13.9%) |
|  | Left  hindfoot | 2 (3.1%) | 10 (15.4%) | 12 (9.2%) |
|  | Right forefoot | 19 (29.2%) | 17 (26.1%) | 36 (27.7%) |
|  | Right midfoot | 13 (20.0%) | 8 (12.3%) | 21 (16.1%) |
|  | Right  hindfoot | 7 (10.8%) | 3 (4.6%) | 10 (7.7%) |
| Number of weeks trial ulcer unhealed | n | 65 | 65 | 130 |
|  | Mean (SD) | 53.5 (105.3) | 48.0 (68.9) | 50.8 (88.7) |
|  | Min - Max | 7.0 – 500.0 | 6.0 – 469.0 | 6.0 – 500.0 |
|  | Median | 22.0 | 26.0 | 26.0 |
|  | P_25_ - P_75_ | 13.0 – 44.0 | 13.0 – 52.0 | 13.0 – 52.0 |
| Is the trial ulcer recurrent? | Yes | 46 (70.8%) | 35 (53.9%) | 81 (62.3%) |
|  | No | 19 (29.2%) | 30 (46.1%) | 49 (37.7%) |
| **Among those whose trial ulcer is recurrent** | |  |  |  |
| Time that the recurrent ulcer has been present in weeks | n | 46 | 35 | 81 |
|  | Mean (SD) | 368 (522) | 416 (589) | 389 (549) |
|  | Min - Max | 9 – 2004 | 12 – 2004 | 9 – 2004 |
|  | Median | 172 | 100 | 148 |
|  | P_25_ - P_75_ | 52 – 420 | 36 – 580 | 49 – 420 |
| **Manual measurements of the trial ulcer (for eligibility) ^4^** | | |  |  |
| Trial ulcer Max Length (cm) | n | 65 | 65 | 130 |
|  | Mean (SD) | 2.8 (1.1) | 2.7 (1.1) | 2.8 (1.1) |
|  | Min - Max | 1.6 – 6.0 | 1.4 – 6.0 | 1.4 – 6.0 |
| Trial ulcer Max Breadth (cm) | n | 65 | 65 | 130 |
|  | Mean (SD) | 2.1 (0.7) | 1.9 (0.5) | 2.0 (0.7) |
|  | Min - Max | 1.0 – 4.2 | 1.0 – 3.3 | 1.0 – 4.2 |
| Trial ulcer Area (cm^2^) | n | 65 | 65 | 130 |
|  | Mean (SD) | 6.1 (4.2) | 5.5 (3.7) | 5.8 (4.0) |
|  | Min - Max | 2.0 – 19.1 | 2.0 – 15.4 | 2.0 – 19.1 |
| **ARANZ manual baseline measurements of the trial ulcer^4^** | | | | |
| Trial ulcer Max Length (cm) | n | 65 | 65 | 130 |
|  | Mean (SD) | 3.0 (1.4) | 2.8 (1.2) | 2.9 (1.3) |
|  | Min - Max | 1.2 – 7.9 | 0.8 – 6.1 | 0.8 – 7.9 |
| Trial ulcer Max Breadth (cm) | n | 65 | 65 | 130 |
|  | Mean (SD) | 2.0 (0.8) | 1.9 (0.7) | 1.9 (0.8) |
|  | Min - Max | 0.8 – 4.9 | 0.5 – 3.9 | 0.5 – 4.9 |
| Trial ulcer Area (cm^2^) | n | 65 | 65 | 130 |
|  | Mean (SD) | 4.8 (4.0) | 4.1 (3.0) | 4.4 (3.5) |
|  | Min - Max | 0.8 – 17.9 | 0.3 – 12.9 | 0.3 – 17.9 |
| **ARANZ automated baseline measurements of the trial ulcer^4^** | | | | |
| Trial ulcer Max Length (cm) | n | 63 | 62 | 125 |
|  | Mean (SD) | 3.0 (1.2) | 2.9 (1.2) | 2.9 (1.2) |
|  | Min - Max | 1.4 – 6.6 | 1.2 – 6.2 | 1.2 – 6.6 |
|  | Missing | 2 | 3 | 5 |
| Trial ulcer Max Breadth (cm) | n | 63 | 62 | 125 |
|  | Mean (SD) | 2.0 (0.7) | 1.9 (0.7) | 1.9 (0.7) |
|  | Min - Max | 0.9 – 4.3 | 0.8 – 3.5 | 0.8 – 4.3 |
|  | Missing | 2 | 3 | 5 |
| Trial ulcer Area (cm^2^) | n | 63 | 62 | 125 |
|  | Mean (SD) | 4.4 (3.4) | 4.0 (2.7) | 4.2 (3.1) |
|  | Min - Max | 1.1 – 16.1 | 0.8 – 11.6 | 0.8 – 16.1 |
|  | Missing | 2 | 3 | 5 |
| **PUSH baseline measurements of the trial ulcer^4^** | | | | |
| Trial ulcer Max Length (cm) | n | 65 | 64 | 129 |
|  | Mean (SD) | 2.6 (1.2) | 2.4 (1.2) | 2.5 (1.2) |
|  | Min - Max | 1.1 – 6.8 | 0.7 – 6.6 | 0.7 – 6.8 |
|  | Missing | 0 | 1 | 1 |
| Trial ulcer Max Breadth (cm) | n | 65 | 64 | 129 |
|  | Mean (SD) | 1.8 (0.7) | 1.9 (1.1) | 1.8 (0.9) |
|  | Min - Max | 0.4 – 3.6 | 0.6 – 7.8 | 0.4 – 7.8 |
|  | Missing | 0 | 1 | 1 |
| Trial ulcer Area^1^ (cm^2^) | n | 65 | 64 | 129 |
|  | Mean (SD) | 3.9 (3.0) | 3.7 (2.8) | 3.8 (2.9) |
|  | Min - Max | 0.6 – 12.5 | 0.9 – 11.3 | 0.6 – 12.5 |
|  | Missing | 0 | 1 | 1 |

*Data are either mean (SD) or number (%)*

*1: Only the area of the trial ulcer was used in covariate adjustment.*

*2: Either participant’s date of birth is provided and the age at randomisation was calculated using the date of randomisation or the age at randomisation is estimated.*

*3: Other diseases in Dressing Changes with Normal Saline Group were: Hypertension (n=4), Hypertension under treatment (n=4); and other diseases in Dressing Changes with L-PRF Matrix Group were: Hypertension (n=2), Hypertension under treatment (n=9), Thyroid under medication(n=1).*

*4: Baseline ulcers were assessed with 4 different methods: (1) manually for assessing eligibility, (2) manually using the ARANZ tool, (3) automatically using the ARANZ tool, and (4) manually using the PUSH tool. The PUSH measurements were used in covariate adjustments.*
