# Supplementary material for: A randomised Trial of Autologous Blood products, leukocyte and platelet-rich fibrin (L-PRF), to promote ulcer healing in LEprosy: The TABLE trial
Source: PLoS Negl Trop Dis. 2024 May 2;18(5):e0012088. doi: 10.1371/journal.pntd.0012088 (PMC11093377; doi:10.1371/journal.pntd.0012088)
Supplement: S6 Table — (DOCX) [file pntd.0012088.s006.docx]

**S6 Table.** Adherence to allocated intervention measured at 42 days

|  | **Allocated intervention** | |
| --- | --- | --- |
|  | **Dressing changes with normal saline^1^ (N=65)** | **Dressing changes with L-PRF matrix**  **(N=65)** |
| Number of participants always received randomised allocation until their ulcer healed | 65 (100%) | 63 (96.9%) |
| Number of participants received alternate allocation at least once | 0 (0%) | 2 (3.1%) ^2^ |
| Number of participants received no intervention at least once | 0 (0%) | 0 (0%) |
| 75% ≤ adherence rate ^3^ < 100% | 0 (0%) | 2 (3.1%) |
| 50% ≤ adherence rate ^3^ < 75% | 0 (0%) | 0 (0%) |
| 25% ≤ adherence rate ^3^ < 50% | 0 (0%) | 0 (0%) |
| 0% ≤ adherence rate ^3^ < 25% | 0 (0%) | 0 (0%) |

*1: If a participant in the dressing changes with normal saline group had an unhealed ulcer at 42 days and the responsible clinician felt that progress was slow and the participant wished, then the participant could transfer to receive the intervention.*

*2: One participant had fever and did not receive an L-PRF matrix change in two dressing changes, and one tested positive for COVID-19, was transferred to another ward, and therefore did not receive an L-PRF matrix in two dressing changes.*

*3: The adherence rate was calculated using the formula: (Number of dressing change forms where participant received randomised allocation / Total number of dressing change forms until 42 days or discharge (whichever is earlier))*100.*
