# Supplementary material for: A randomised Trial of Autologous Blood products, leukocyte and platelet-rich fibrin (L-PRF), to promote ulcer healing in LEprosy: The TABLE trial
Source: PLoS Negl Trop Dis. 2024 May 2;18(5):e0012088. doi: 10.1371/journal.pntd.0012088 (PMC11093377; doi:10.1371/journal.pntd.0012088)
Supplement: S7 Table — (DOCX) [file pntd.0012088.s007.docx]

**S7 Table.** P-Values adjustment for multiple testing (ITT analysis)

| **Outcome** | **Initial**  **p-value** | **Adjusted^1^**  **p-value** |
| --- | --- | --- |
| **Rate of healing** | | |
| Unadjusted mixed effects regression model for ARANZ auto-measurements | 0.027 | 0.083 |
| Adjusted^2^ mixed effects regression model for ARANZ auto-measurements | 0.028 | 0.083 |
| Unadjusted mixed effects regression model for ARANZ manual-measurements | 0.008 | 0.044 |
| Adjusted^2^ mixed effects regression model for ARANZ manual-measurements | 0.009 | 0.044 |
| Unadjusted mixed effects model for PUSH measurements | 0.313 | 0.323 |
| Adjusted^2^ mixed effects regression model for PUSH measurements | 0.323 | 0.323 |
| **Complete re-epithelialisation** | | |
| Unadjusted cox proportional hazards regression model using clinical assessment | 0.2997 | 0.4615 |
| Adjusted^3^ cox proportional hazards regression model using clinical assessment | 0.2064 | 0.4615 |
| Unadjusted cox proportional hazards regression model using blinded assessor’s assessment | 0.4615 | 0.4615 |
| Adjusted^4^ cox proportional hazards regression model using blinded assessor’s assessment | 0.3698 | 0.4615 |

*1: P-values were adjusted for multiple testing using the Hochberg stepdown method.*

*2: Adjusted for the baseline value of participant age and including the interaction terms between time and treatment, and time^2 and treatment. Baseline participants’ age was treated as a continuous variable and considered as a fixed effect in this adjustment.*

*3: Adjusted for the baseline values of trial ulcer size and participants’ age. Trial ulcer size and participants’ age were both treated as continuous variables and considered as fixed effects in this adjustment.*
