# Supplementary material for: A randomised Trial of Autologous Blood products, leukocyte and platelet-rich fibrin (L-PRF), to promote ulcer healing in LEprosy: The TABLE trial
Source: PLoS Negl Trop Dis. 2024 May 2;18(5):e0012088. doi: 10.1371/journal.pntd.0012088 (PMC11093377; doi:10.1371/journal.pntd.0012088)
Supplement: S9 Table — (DOCX) [file pntd.0012088.s009.docx]

**S9 Table.** Analysis of primary outcome measure – Time to complete re-epithelisation censored at 42 days (Per-protocol (adherent) analysis)

|  | **Dressing changes with normal saline (n=65)** | **Dressing changes with L-PRF matrix (n=63)** | **Unadjusted Hazard Ratio^2^**  **(95% CI)**  **p-value** | **Adjusted^1^**  **Hazard Ratio^2^**  **(95% CI)**  **p-value** |
| --- | --- | --- | --- | --- |
| Healing assessed by clinician | | | | |
| Number of censored participants | 33 (50.8%) | 27 (42.9%)^3^ | 1.4  (0.8 to 2.2)  p=0.218 | 1.4  (0.9 to 2.3)  p=0.161 |
| Number of healed participants | 32 (49.2%) | 36 (57.1%) |  |  |

*1: Cox proportional hazards adjusted for the baseline values of trial ulcer size and participant age. Trial ulcer size and participant age were treated as continuous variables and considered as fixed effects in this adjustment.*

*2: HR>1 means – Participants in Dressing Changes with L-PRF Matrix Group are more likely to have completely re-epithelialised ulcers than participants in Dressing Changes with Normal Saline Group.*

*3: Two participants were excluded from the per protocol analysis because they were considered as non-adherence.*
