# Supplementary material for: A randomised Trial of Autologous Blood products, leukocyte and platelet-rich fibrin (L-PRF), to promote ulcer healing in LEprosy: The TABLE trial
Source: PLoS Negl Trop Dis. 2024 May 2;18(5):e0012088. doi: 10.1371/journal.pntd.0012088 (PMC11093377; doi:10.1371/journal.pntd.0012088)
Supplement: S10 Table — (DOCX) [file pntd.0012088.s010.docx]

**S****10 Table.** Mean difference in daily healing rate over 42 days for each model - Missing data analysis

|  | **Unadjusted Model^1,2^** | **Adjusted Model^3,2^** |
| --- | --- | --- |
|  | **Average difference**  **(95% CI^4^)**  **p-value** | **Average difference**  **(95% CI^4^)**  **p-value** |
| ARANZ auto tool, cm^2^ | 0.011 (0.0007 to 0.022)  p=0.037 | 0.011 (0.0006 to 0.022)  p=0.039 |
| ARANZ manual tool, cm^2^ | 0.016 (0.004 to 0.028)  p=0.007 | 0.016 (0.004 to 0.027)  p=0.008 |
| PUSH tool, cm^2^ | 0.005 (-0.005 to 0.016)  p=0.306 | 0.005 (-0.005 to 0.016)  p=0.315 |

*1: Unadjusted mixed effects regression model with time modelled time as a quadratic function. Model includes interaction terms between time and treatment and time^2 and treatment.*

*2: Estimated average difference>0 indicates a higher daily healing rate in dressing change with L-PRF matrix group.*

*3: Mixed effects regression model adjusted for the baseline value of participant age, with time modelled as a quadratic function. Model includes the interaction terms between time and treatment, and time^2 and treatment. Baseline participant age was treated as a continuous variable and considered as a fixed effect in this adjustment.*

*4: Using the Wald statistics to estimate the confidence interval.*
