# Supplementary material for: A randomised Trial of Autologous Blood products, leukocyte and platelet-rich fibrin (L-PRF), to promote ulcer healing in LEprosy: The TABLE trial
Source: PLoS Negl Trop Dis. 2024 May 2;18(5):e0012088. doi: 10.1371/journal.pntd.0012088 (PMC11093377; doi:10.1371/journal.pntd.0012088)
Supplement: S11 Table — (DOCX) [file pntd.0012088.s011.docx]

**S11 Table.** Baseline characteristics of those who provided data that allows the rate of healing outcome using the ARANZ automated method to be assessed, vs. those who did not.

|  |  | **Participants who provided data**  **(N=83)** | **Participants who did not provide data**  **(N=47)** |
| --- | --- | --- | --- |
| **Variables used in covariate adjustment** | |  |  |
| Trial ulcer Area^1^ (cm^2^) -PUSH tool | n | 82 | 47 |
|  | Mean (SD) | 4.0 (2.9) | 3.5 (2.8) |
|  | Min - Max | 1.0 – 12.5 | 0.6 – 10.9 |
|  | Missing | 1 | 0 |
| Date of birth known? | Yes | 33 (39.8%) | 23 (48.9%) |
|  | No | 50 (60.2%) | 24 (51.1%) |
| Age at randomisation (years)^2^ | n | 83 | 47 |
|  | Mean (SD) | 53.0 (16.2) | 55.8 (15.0) |
|  | Min - Max | 20.0 – 84.0 | 22.0 – 89.0 |
| **Participant demographics** | |  |  |
| Gender | Male | 68 (81.9%) | 35 (74.5%) |
|  | Female | 15 (18.1%) | 12 (25.5%) |
|  | Other | 0 (0%) | 0 (0%) |
| Highest level of education | Never joined formal school | 30 (36.1%) | 21 (44.7%) |
|  | Can read and write | 16 (19.3%) | 7 (14.9%) |
|  | Primary level | 21 (25.3%) | 6 (12.8%) |
|  | Secondary level | 15 (18.1%) | 10 (21.3%) |
|  | Higher secondary level | 1 (1.2%) | 3 (6.3%) |
|  | University level | 0 (0%) | 0 (0%) |
| **Clinical information** |  |  |  |
| Height in cm | n | 83 | 47 |
|  | Mean (SD) | 158.6 (7.4) | 155.4 (9.0) |
|  | Min - Max | 141.0 – 178.0 | 136.5 – 172.5 |
| Weight in kg | n | 83 | 47 |
|  | Mean (SD) | 56.1 (9.6) | 55.0 (8.6) |
|  | Min - Max | 37.5 – 85.3 | 30.0 – 80.9 |
| BMI | n | 83 | 47 |
|  | Mean (SD) | 22.3 (3.4) | 22.9 (3.7) |
|  | Min - Max | 16.9 – 32.0 | 14.9 – 32.5 |
| Blood Pressure – Systolic (mmHg) | n | 83 | 47 |
|  | Mean (SD) | 111.2 (9.8) | 117.4 (11.7) |
|  | Min - Max | 90.0 – 140.0 | 90.0 – 150.0 |
| Blood Pressure – Diastolic (mmHg) | n | 83 | 47 |
|  | Mean (SD) | 76.9 (8.1) | 78.9 (7.6) |
|  | Min - Max | 50.0 – 90.0 | 70.0 – 100.0 |
| Platelet result x 10^3^/ul | n | 83 | 47 |
|  | Mean (SD) | 287.8 (89.5) | 276.4 (86.9) |
|  | Min - Max | 128.0 – 572.0 | 127.0 – 529.0 |
| Haemoglobin result in gm/dL | n | 83 | 47 |
|  | Mean (SD) | 13.6 (1.9) | 13.8 (1.8) |
|  | Min - Max | 9.7 – 18.8 | 10.0 – 18.1 |
|  | Missing | 0 | 0 |
| Fasting blood sugar result in mg/dL | n | 83 | 47 |
|  | Mean (SD) | 83.0 (11.2) | 86.8 (12.5) |
|  | Min - Max | 50.0 – 110.0 | 60.0 – 109.0 |
| **Leprosy details** |  |  |  |
| Number of years since leprosy diagnosis | n | 83 | 47 |
|  | Mean (SD) | 19.2 (14.8) | 20.7 (14.7) |
|  | Min - Max | 1.0 – 60.0 | 1.0 – 58.0 |
| Oral antibiotic (multi-drug therapy) treatment for leprosy | Ongoing | 10 (12.1%) | 1 (2.1%) |
|  | Completed | 73 (87.1%) | 45 (95.7%) |
|  | Unknown | 0 (0%) | 1 (2.1%) |
|  |  |  |  |
| Diseases other than leprosy^3^ | Yes | 14 (16.9%) | 6 (12.8%) |
|  | No | 69 (83.1%) | 41 (87.2%) |
| **VMT/ST** |  |  |  |
| VMT/ST | Normal | 0 (0%) | 0 (0%) |
|  | Impaired | 83 (100%) | 47 (100%) |
| **Among Impaired** |  |  |  |
| Any nerve enlarged in leg | Left | 21 (25.3%) | 19 (40.4%) |
|  | Right | 21 (25.3%) | 15 (31.9%) |
|  | Nil | 41 (49.4%) | 13 (27.7%) |
| Any loss of sensation in foot | Left | 37 (44.6%) | 26 (55.3%) |
|  | Right | 46 (55.4%) | 21 (44.7%) |
|  | Nil | 0 (0%) |  |
| Any loss of motor function in foot | Left | 19 (22.9%) | 13 (27.7%) |
|  | Right | 24 (28.9%) | 10 (21.3%) |
|  | Nil | 40 (48.2%) | 24 (51.0%) |
|  | Missing | 0 | 0 |
| Any deformity in foot | Left | 32 (38.6%) | 23 (48.9%) |
|  | Right | 43 (51.8%) | 19 (40.4%) |
|  | Nil | 8 (9.6%) | 5 (10.7%) |
| **Current Ulcer Information** | |  |  |
| Total number of current ulcers, Left foot | n | 83 | 47 |
|  | Mean (SD) | 0.5 (0.6) | 0.6 (0.6) |
|  | Min - Max | 0.0 – 2.0 | 0.0 – 2.0 |
|  | 0 ulcers | 44 (53.0%) | 20 (42.6%) |
|  | 1 ulcer | 35 (42.2%) | 24 (51.0%) |
|  | 2 ulcers | 4 (4.8%) | 3 (6.4%) |
| Total number of current ulcers, Right foot | n | 83 | 47 |
|  | Mean (SD) | 0.7 (0.6) | 0.6 (0.6) |
|  | Min - Max | 0.0 – 2.0 | 0.0 – 2.0 |
|  | 0 ulcers | 32 (38.6%) | 22 (46.8%) |
|  | 1 ulcer | 42 (50.6%) | 23 (48.9%) |
|  | 2 ulcers | 9 (10.8%) | 2 (4.3%) |
| Location of the trial ulcer | Left hindfoot | 6 (7.2%) | 6 (12.8%) |
|  | Left forefoot | 19 (22.9%) | 14 (29.8%) |
|  | Left midfoot | 12 (14.5%) | 6 (12.8%) |
|  | Right midfoot | 17 (20.5%) | 4 (8.5%) |
|  | Right forefoot | 25 (30.1%) | 11 (23.4%) |
|  | Right hindfoot | 4 (4.8%) | 6 (12.8%) |
| Number of weeks trial ulcer unhealed | n | 83 | 47 |
|  | Mean (SD) | 40.8 (64.9) | 68.3 (118.5) |
|  | Min - Max | 6.0 – 500.0 | 6.0 – 500.0 |
|  | Median | 26.0 | 26.0 |
|  | P_25_ – P_75_ | 15.0 – 35.0 | 13.0 – 52.0 |
| Is the trial ulcer recurrent? | Yes | 48 (57.8%) | 33 (70.2%) |
|  | No | 35 (42.2%) | 14 (29.8%) |
| **Among those whose trial ulcer is recurrent** | |  |  |
| Time that the recurrent ulcer has been present in weeks | n | 48 | 33 |
|  | Mean (SD) | 512.3 (613.0) | 209.2 (381.7) |
|  | Min - Max | 24.0 – 2004.0 | 9.0 – 1821.0 |
|  | Median | 302.0 | 96.0 |
|  | P_25_ – P_75_ | 60.0 – 580.0 | 28.0 – 148.0 |
| **Manual measurements of the trial ulcer (for eligibility) ^4^** | | |  |
| Trial ulcer Max Length (cm) | n | 83 | 47 |
|  | Mean (SD) | 2.8 (1.1) | 2.7 (1.0) |
|  | Min - Max | 1.4 – 6.0 | 1.4 – 5.5 |
| Trial ulcer Max Breadth (cm) | n | 83 | 47 |
|  | Mean (SD) | 2.0 (0.6) | 1.9 (0.7) |
|  | Min - Max | 1.0 – 4.0 | 1.0 – 4.2 |
| Trial ulcer Area (cm^2^) | n | 83 | 47 |
|  | Mean (SD) | 6.0 (4.1) | 5.5 (3.8) |
|  | Min - Max | 2.0 – 19.1 | 2.0 – 18.9 |
| **ARANZ manual baseline measurements of the trial ulcer^4^** | | | |
| Trial ulcer Max Length (cm) | n | 83 | 47 |
|  | Mean (SD) | 3.0 (1.3) | 2.8 (1.2) |
|  | Min - Max | 1.2 – 7.9 | 0.8 – 6.1 |
| Trial ulcer Max Breadth (cm) | n | 83 | 47 |
|  | Mean (SD) | 2.0 (0.7) | 1.9 (0.8) |
|  | Min - Max | 0.8 – 4.3 | 0.5 – 4.9 |
| Trial ulcer Area (cm^2^) | n | 83 | 47 |
|  | Mean (SD) | 4.6 (3.5) | 4.2 (3.5) |
|  | Min - Max | 0.8 – 17.6 | 0.3 – 17.9 |
| **ARANZ automated baseline measurements of the trial ulcer^4^** | | | |
| Trial ulcer Max Length (cm) | n | 83 | 47 |
|  | Mean (SD) | 2.9 (1.2) | 2.9 (1.2) |
|  | Min - Max | 1.2 – 6.6 | 1.5 – 6.2 |
| Trial ulcer Max Breadth (cm) | n | 83 | 47 |
|  | Mean (SD) | 1.9 (0.7) | 1.9 (0.7) |
|  | Min - Max | 0.8 – 4.3 | 1.1 – 4.2 |
| Trial ulcer Area (cm^2^) | n | 83 | 47 |
|  | Mean (SD) | 4.2 (3.1) | 4.2 (3.1) |
|  | Min - Max | 0.8 – 16.1 | 1.0 – 16.1 |
| **PUSH baseline measurements of the trial ulcer^4^** | | | |
| Trial ulcer Max Length (cm) | n | 82 | 47 |
|  | Mean (SD) | 2.6 (1.2) | 2.3 (1.1) |
|  | Min - Max | 1.0 – 6.8 | 0.7 – 5.4 |
|  | Missing | 1 | 0 |
| Trial ulcer Max Breadth (cm) | n | 82 | 47 |
|  | Mean (SD) | 1.9 (0.9) | 1.8 (0.8) |
|  | Min - Max | 0.6 – 7.8 | 0.4 – 4.0 |
|  | Missing | 1 | 0 |
| Trial ulcer Area^1^ (cm^2^) | n | 82 | 47 |
|  | Mean (SD) | 4.0 (2.9) | 3.5 (2.8) |
|  | Min - Max | 1.0 – 12.5 | 0.6 – 10.9 |
|  | Missing | 1 | 0 |

*Data are either mean (SD) or number (%).*

*1: Only the area of the trial ulcer was used in covariate adjustment.*

*2: Either participant’s date of birth is provided and the age at randomisation is calculated using the date of randomisation or the age at randomisation is estimated.*

*3: Other diseases among those who provided data were: Hypertension (n=3), Hypertension under treatment (n=11); and other diseases among those who did not provide data were: Hypertension (n=3), Hypertension under treatment (n=2), Thyroid under medication(n=1).*

*4: Baseline ulcers was assessed with 4 different methods: manually for assessing eligibility, manually using the ARANZ tool, automatically using the ARANZ tool and manually using the PUSH tool. The PUSH measurements were used in covariate adjustments.*
