# Supplementary material for: A randomised Trial of Autologous Blood products, leukocyte and platelet-rich fibrin (L-PRF), to promote ulcer healing in LEprosy: The TABLE trial
Source: PLoS Negl Trop Dis. 2024 May 2;18(5):e0012088. doi: 10.1371/journal.pntd.0012088 (PMC11093377; doi:10.1371/journal.pntd.0012088)
Supplement: S12 Table — (DOCX) [file pntd.0012088.s012.docx]

**S12 Table.** Mean difference in daily healing rate over 42 days for each model – Subgroup analysis

|  |  | **Unadjusted Model^1,2^** | **Adjusted Model^3,2^** |
| --- | --- | --- | --- |
|  |  | **Average difference**  **(95% CI^4^)**  **p-value** | **Average difference**  **(95% CI^4^)**  **p-value** |
| ARANZ auto tool, cm^2^ | Baseline Ulcer Size ≥ Median=2.79 | 0.02 (0.007 to 0.03)  p=0.003 | 0.02 (0.006 to 0.03)  p=0.003 |
|  | Baseline Ulcer Size < Median=2.79 | 0.0008 (-0.01 to 0.02)  p=0.919 | 0.0006 (-0.01 to 0.02)  p=0.937 |
| ARANZ manual tool, cm^2^ | Baseline Ulcer Size ≥ Median=2.79 | 0.02 (0.009 to 0.03)  p=0.001 | 0.02 (0.009 to 0.03)  p=0.001 |
|  | Baseline Ulcer Size < Median=2.79 | 0.0006 (-0.02 to 0.02)  p=0.943 | 0.0004 (-0.02 to 0.02)  p=0.959 |
| PUSH tool, cm^2^ | Baseline Ulcer Size ≥ Median=2.79 | 0.005 (-0.007 to 0.02)  p=0.396 | 0.005 (-0.007 to 0.02)  p=0.410 |
|  | Baseline Ulcer Size < Median=2.79 | -0.002 (-0.02 to 0.01)  p=0.742 | -0.003 (-0.02 to 0.01)  p=0.724 |

*1: Mixed effects regression model adjusted for time, treatment by time, treatment by time^2, treatment by subgroup, treatment by time by subgroup, and treatment by time^2 by subgroup interactions.*

*2: Estimated average difference>0 indicates a higher daily healing rate in dressing change with L-PRF matrix group.*

*3: Mixed effects regression model adjusted for the baseline values of participant age and time, treatment by time, treatment by time^2, treatment by subgroup, treatment by time by subgroup, and treatment by time^2 by subgroup interactions. Baseline participant age was treated as a continuous variable and considered as a fixed effect in this adjustment.*

*4: 95% confidence interval was not possible to be estimated.*
