# Supplementary material for: A randomised Trial of Autologous Blood products, leukocyte and platelet-rich fibrin (L-PRF), to promote ulcer healing in LEprosy: The TABLE trial
Source: PLoS Negl Trop Dis. 2024 May 2;18(5):e0012088. doi: 10.1371/journal.pntd.0012088 (PMC11093377; doi:10.1371/journal.pntd.0012088)
Supplement: S13 Table — (DOCX) [file pntd.0012088.s013.docx]

**S13 Table.** Subgroup analysis of the time to complete re-epithelisation outcome assessed by clinician censored at 42 days and including the area of the baseline ulcer

|  | | | | **Adjusted Model I^1^** | | **Adjusted Model II^2^** | |
| --- | --- | --- | --- | --- | --- | --- | --- |
|  |  | **Dressing changes with normal saline (n=65)** | **Dressing changes with L-PRF matrix (n=65)** | **Unadjusted Hazard Ratio^3^**  **(95% CI)**  **p-value** | **Treatment and**  **subgroup**  **Interaction**  **p-value** | **Adjusted**  **Hazard Ratio^3^**  **(95% CI)**  **p-value** | **Treatment and**  **subgroup**  **Interaction**  **p-value** |
| Ulcer Size ≥ Median=2.79 | N | 36 | 29 | 1.0  (0.5 to 2.1)  p=0.995 | p=0.394 | 1.1  (0.5 to 2.4)  p=0.819 | p=0.501 |
|  | Number of censored participants | 21 (58.3%) | 17 (58.6%) |  |  |  |  |
|  | Number of healed participants | 15 (41.7%) | 12 (41.4%) |  |  |  |  |
| Ulcer Size < Median=2.79 | N | 29 | 35 | 1.5  (0.8 to 2.8)  p=0.181 |  | 1.5  (0.8 to 2.9)  p=0.177 |  |
|  | Number of censored participants | 12 (41.4%) | 11 (31.4%) |  |  |  |  |
|  | Number of healed participants | 17 (58.6%) | 24 (68.6) |  |  |  |  |

*1: Adjusted for treatment by subgroup interaction.*

*2: Adjusted for the baseline values of trial ulcer size, participant age, and treatment by subgroup interactions. Baseline trial ulcer size and participant age were treated as continuous variables and considered as fixed effects in this adjustment.*

*3: HR>1 means – Participants in Dressing Changes with L-PRF Matrix Group are more likely to have completely re-epithelialised ulcers than participants in Dressing Changes with Normal Saline Group.*
