# Supplementary material for: A randomised Trial of Autologous Blood products, leukocyte and platelet-rich fibrin (L-PRF), to promote ulcer healing in LEprosy: The TABLE trial
Source: PLoS Negl Trop Dis. 2024 May 2;18(5):e0012088. doi: 10.1371/journal.pntd.0012088 (PMC11093377; doi:10.1371/journal.pntd.0012088)
Supplement: S14 Table — (DOCX) [file pntd.0012088.s014.docx]

**S14 Table.** Area of ulcer size using each of the three tools at chosen time points censored at 70 days

|  | **Unadjusted Model^1^** | | | | **Adjusted Model^2^** | | | |
| --- | --- | --- | --- | --- | --- | --- | --- | --- |
|  | **Time by Treatment Interaction Estimate**  **(p-value)** | **Time^2 by Treatment Interaction Estimate**  **(p-value)** | **Time point** | **Mean Difference^3^ (95% CI)** | **Time by Treatment Interaction Estimate**  **(p-value)** | **Time^2 by Treatment Interaction Estimate**  **(p-value)** | **Time point** | **Mean Difference^3^ (95% CI)** |
| **ARANZ auto-measurements** | 0.01  (p=0.324) | 0.0001  (p=0.780) | Day:0 | -0.5 (-1.2 to 0.1) | 0.01  (p=0.331) | 0.0001  (p=0.774) | Day:0 | -0.5 (-1.2 to 0.1) |
|  |  |  | Day:10 | -0.4 (-1.0 to 0.2) |  |  | Day:10 | -0.4 (-1.0 to 0.2) |
|  |  |  | Day:23 | -0.3 (-0.9 to 0.4) |  |  | Day:23 | -0.3 (-0.9 to 0.3) |
|  |  |  | Day:35 | -0.1 (-0.7 to 0.5) |  |  | Day:35 | -0.1 (-0.7 to 0.5) |
|  |  |  | Day:42 | 0.01 (-0.6 to 0.6) |  |  | Day:42 | -0.01 (-0.6 to 0.6) |
|  |  |  | Day:55 | 0.2 (-0.5 to 0.9) |  |  | Day:55 | 0.2 (-0.5 to 0.9) |
|  |  |  | Day:70 | 0.5 (-0.4 to 1.3) |  |  | Day:70 | 0.5 (-0.4 to 1.3) |
| **ARANZ manual-measurements** | 0.02  (p=0.165) | 0.000002  (p=0.992) | Day:0 | -0.7 (-1.4 to 0.001) | 0.02  (p=0.170) | 0.000003  (p=0.987) | Day:0 | -0.7 (-1.4 to -0.03) |
|  |  |  | Day:10 | -0.5 (-1.2 to 0.1) |  |  | Day:10 | -0.6 (-1.2 to 0.1) |
|  |  |  | Day:23 | -0.3 (-1.0 to 0.3) |  |  | Day:23 | -0.4 (-1.0 to 0.3) |
|  |  |  | Day:35 | -0.2 (-0.8 to 0.5) |  |  | Day:35 | -0.2 (-0.9 to 0.5) |
|  |  |  | Day:42 | -0.04 (-0.7 to 0.7) |  |  | Day:42 | -0.1 (-0.7 to 0.6) |
|  |  |  | Day:55 | 0.2 (-0.6 to 0.9) |  |  | Day:55 | 0.1 (-0.6 to 0.9) |
|  |  |  | Day:70 | 0.4 (-0.5 to 1.3) |  |  | Day:70 | 0.4 (-0.5 to 1.3) |
| **PUSH measurements** | -0.01  (p=0.459) | 0.0002  (p=0.127) | Day:0 | -0.2 (-0.8 to 0.4) | -0.01  (p=0.450) | 0.0002  (p=0.125) | Day:0 | -0.2 (-0.8 to 0.3) |
|  |  |  | Day:10 | -0.3 (-0.8 to 0.3) |  |  | Day:10 | -0.3 (-0.8 to 0.3) |
|  |  |  | Day:23 | -0.3 (-0.8 to 0.3) |  |  | Day:23 | -0.3 (-0.8 to 0.3) |
|  |  |  | Day:35 | -0.2 (-0.7 to 0.4) |  |  | Day:35 | -0.2 (-0.8 to 0.4) |
|  |  |  | Day:42 | -0.1 (-0.7 to 0.5) |  |  | Day:42 | -0.1 (-0.7 to 0.4) |
|  |  |  | Day:55 | 0.1 (-0.5 to 0.7) |  |  | Day:55 | 0.1 (-0.5 to 0.7) |
|  |  |  | Day:70 | 0.5 (-0.3 to 1.2) |  |  | Day:70 | 0.5 (-0.3 to 1.2) |

*1: Unadjusted mixed effects regression model including the interaction terms between time and treatment and time^2 and treatment; and modelling time as Quadratic Predictor.*

*2: Mixed effects regression model adjusted for the baseline value of participant age and including the interaction terms between time and treatment, and time^2 and treatment; and modelling time as Quadratic Predictor. Baseline participant age was treated as a continuous variable and considered as a fixed effect in this adjustment.*

*3: Values of means differences<0 indicate a larger average ulcer size area in Dressing Changes with normal saline Group.*
