# Supplementary material for: A randomised Trial of Autologous Blood products, leukocyte and platelet-rich fibrin (L-PRF), to promote ulcer healing in LEprosy: The TABLE trial
Source: PLoS Negl Trop Dis. 2024 May 2;18(5):e0012088. doi: 10.1371/journal.pntd.0012088 (PMC11093377; doi:10.1371/journal.pntd.0012088)
Supplement: S15 Table — (DOCX) [file pntd.0012088.s015.docx]

**S15 Table.** Analysis of the time to complete re-epithelisation outcome censored at 70 days (ITT analysis)

|  | **Dressing changes with normal saline (n=65)** | **Dressing changes with L-PRF matrix (n=65)** | **Unadjusted Hazard Ratio ^1^**  **(95% CI)**  **p=value** | **Adjusted^2^**  **Hazard Ratio ^1^**  **(95% CI)**  **p=value** | **Adjusted^3,4^**  **Hazard Ratio^1^**  **(95% CI)**  **p=value** | **Adjusted^3,5^**  **Hazard Ratio^1^**  **(95% CI)**  **p=value** | **Adjusted^3,6^**  **Hazard Ratio^1^**  **(95% CI)**  **p=value** |
| --- | --- | --- | --- | --- | --- | --- | --- |
| Healing assessed by clinician | | | | | | | |
| Number of censored participants^7^ | 12 (18.5%) | 9 (13.8%) | 1.4 (0.9 to 2.0)  p=0.118 | 1.5 (1.0 to 2.2)  p=0.044 | 1.5 (1.0 to 2.2)  p=0.042 | 1.5 (1.0 to 2.2)  p=0.058 | 1.5 (1.0 to 2.1)  p=0.057 |
| Number of healed participants | 53 (81.5%) | 56 (86.2%) |  |  |  |  |  |

*1: HR>1 means - Participants in Dressing Changes with L-PRF Matrix Group are more likely to have completely re-epithelialised ulcers than participants in Dressing Changes with Normal Saline Group.*

*2: Cox proportional hazard model adjusted for the baseline values of trial ulcer size and participant age. Trial ulcer size and participant age was treated as continuous variables and considered as fixed effects in this adjustment.*

*3: The assumption of proportional hazards was violated for the covariate-baseline ulcer area and thus we conducted extra analysis to assess the impact of this violation.*

*4: Cox proportional hazard model adjusted for only participant age at baseline, which was treated as a continuous variable and considered as a fixed effect in this adjustment. Baseline ulcer measurement was excluded from the model.*

*5: Stratified cox proportional hazard model adjusted for the baseline values of trial ulcer size and participant age. Trial ulcer size was automatically categorised in to four strata. Participant age was treated as a continuous variable and considered as a fixed effect in this adjustment.*

*6: Stratified cox proportional hazard model adjusted for the baseline values of trial ulcer size and participant age. Trial ulcer size was automatically categorised in to two strata. Participant age was treated as a continuous variable and considered as a fixed effect in this adjustment.*

*7: One participant in the dressing changes with normal saline group and 3 participants in the dressing changes with L-PRF matrix group withdrew before having reached 70 days post-randomisation.*
