# Supplementary material for: A randomised Trial of Autologous Blood products, leukocyte and platelet-rich fibrin (L-PRF), to promote ulcer healing in LEprosy: The TABLE trial
Source: PLoS Negl Trop Dis. 2024 May 2;18(5):e0012088. doi: 10.1371/journal.pntd.0012088 (PMC11093377; doi:10.1371/journal.pntd.0012088)
Supplement: S16 Table — (DOCX) [file pntd.0012088.s016.docx]

**S16 Table.** Analysis of continuous secondary outcome- Quality of life ‘area under the curve’ until discharge or 70 days post randomisation, whichever occurred first

|  | **Dressing changes with normal saline (N=65)** | **Dressing changes with L-PRF matrix (N=65)** | **Adjusted Mean Difference^1^**  **(95% CI)**  **p-value** |
| --- | --- | --- | --- |
| **Quality of life ‘area under the curve’ until discharge or 70 days post randomisation, whichever occurs first** | | | |
| N | 65 | 64^2^ | 0.04 (-0.02 to 0.09)  p=0.216 |
| Mean (SD) | 0.52 (0.17) | 0.55 (0.17) |  |
| Min - Max | 0.05 – 0.78 | 0.18 – 0.84 |  |

*1: Linear regression model adjusted for the baseline values of trial ulcer size and participant age. Trial ulcer size and participant age were treated as continuous variables and considered as fixed effects in this adjustment. Adjusted MD > 0 means higher quality of life score during six months was observed in the L-PRF group.*

*2: One baseline measurement of the ulcer area is missing because the photograph could not be calibrated hence all adjustments analyses using the baseline ulcer size have one participant less.*
