# Supplementary material for: A randomised Trial of Autologous Blood products, leukocyte and platelet-rich fibrin (L-PRF), to promote ulcer healing in LEprosy: The TABLE trial
Source: PLoS Negl Trop Dis. 2024 May 2;18(5):e0012088. doi: 10.1371/journal.pntd.0012088 (PMC11093377; doi:10.1371/journal.pntd.0012088)
Supplement: S17 Table — (DOCX) [file pntd.0012088.s017.docx]

**S17 Table.** Recurrence of treated ulcer 6 months from randomisation

|  | **Dressing changes with normal saline (N=65)** | **Dressing changes with L-PRF matrix (N=65)** | **Adjusted Relative Risk^1^**  **(95% CI)**  **p-value** | **Adjusted Risk Difference^2^**  **(95% CI)**  **p-value** |
| --- | --- | --- | --- | --- |
| **Recurrence of treated ulcer at 6 months from randomisation** | | | | |
| Yes | 22 (37.3%) | 18 (30.0%) | 0.80  (0.49 to 1.30)  p=0.367 | -0.05  (-0.22 to 0.11)  p=0.516 |
| No | 37 (62.7%) | 42 (70.0%) |  |  |
| Missing | 1 | 5 |  |  |
| Excluded from analysis | 5 | 0 |  |  |
| **Appearance of a new ulcer at 6 months from randomisation ^3^** | | | | |
| Yes | 6 (10.5%) | 8 (13.6%) | 1.27  (0.48 to 3.40)  p=0.628 | 0.04  (-0.08 to 0.16)  p=0.495 |
| No | 51 (89.5%) | 51 (86.4%) |  |  |
| Missing | 3 | 6 |  |  |
| Excluded from analysis | 5 | 0 |  |  |

*1: Log-binomial regression model adjusted for the baseline values of trial ulcer size and participant age. Trial ulcer size and participant age were treated as continuous variables and considered as fixed effects in this adjustment. Adjusted RR > 1 means a higher rate of recurrent or new ulcers respectively was observed in the L-PRF group.* *The Wald statistic was used to produce the 95% CI.*

*2: Log-binomial regression model using the identity link function adjusted for the baseline values of trial ulcer size and participant age. Trial ulcer size and participant age were treated as continuous variables and considered as fixed effects in this adjustment. Adjusted RD > 0 means higher risk of recurrent or new ulcers respectively for the L-PRF group. The Wald statistics was used to produce the 95% CI.*

*3: The question related to appearance of a new ulcer was included on the 6 months follow up form after the commencement of the trial, therefore responses on this question were not collected for three participants.*
