# Supplementary material for: A randomised Trial of Autologous Blood products, leukocyte and platelet-rich fibrin (L-PRF), to promote ulcer healing in LEprosy: The TABLE trial
Source: PLoS Negl Trop Dis. 2024 May 2;18(5):e0012088. doi: 10.1371/journal.pntd.0012088 (PMC11093377; doi:10.1371/journal.pntd.0012088)
Supplement: S18 Table — (DOCX) [file pntd.0012088.s018.docx]

**S18 Table.** Analyses of activity measurement at 7-, 14-, and 42-days post-randomisation

|  |  | **Dressing changes**  **with normal saline**  **(N=65)** | **Dressing changes with**  **L-PRF matrix**  **(N=65)** | **Mean Difference^1^**  **95% CI**  **p-value** |
| --- | --- | --- | --- | --- |
| Number of participants their steps have not been recorded | N | 21 | 20 | NA |
| Average daily steps measured  at 7 days post-randomisation | N | 44 | 45 | 119.3 (-271.8 to 510.4)  p=0.546 |
|  | Mean (SD) | 1336.4 (916.2) | 1217.1 (939.7) |  |
|  | Min – Max | 174.9 – 4534.6 | 134.8 – 3817.6 |  |
|  | Missing | 0 | 0 |  |
| Average daily steps measured  at 14 days post-randomisation | N | 44 | 45 | 173.4 (-248.2 to 595.1)  p=0.416 |
|  | Mean (SD) | 1502.5 (1040.0) | 1329.0 (960.4) |  |
|  | Min - Max | 223.5 – 5125.9 | 138.9 – 3918.1 |  |
|  | Missing | 0 | 0 |  |
| Average daily steps measured  at 42 days post-randomisation | N | 45 ^2^ | 45 | 173.1 (-302.1 to 648.2)  p=0.471 |
|  | Mean (SD) | 1721.0 (1210.9) | 1547.9 (1051.7) |  |
|  | Min - Max | 214.9 – 6261.2 | 138.9 – 4092.4 |  |
|  | Missing | 0 | 0 |  |

*1: Mean difference is estimated using a t-test. MD>0 indicates higher mean daily steps in normal saline group.*

*2: For one participant only the total number of steps made during the first 42 days was collected and thus this participant is included in the outcome at 42 days only.*
