# Supplementary figures and images for: A randomised Trial of Autologous Blood products, leukocyte and platelet-rich fibrin (L-PRF), to promote ulcer healing in LEprosy: The TABLE trial
Source: PLoS Negl Trop Dis. 2024 May 2;18(5):e0012088. doi: 10.1371/journal.pntd.0012088 (PMC11093377; doi:10.1371/journal.pntd.0012088)

**S1 Figure.** Average duration of a dressing change over time for each group

**
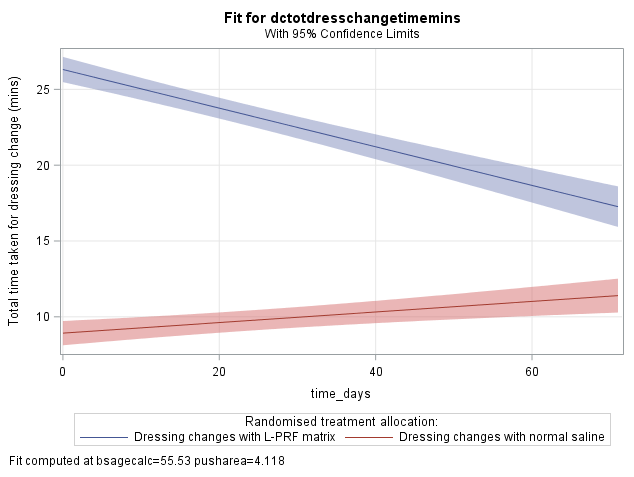
**

Supplement: S1 Fig — (DOCX) [file pntd.0012088.s019.docx]
