# Supplementary material for: A randomised Trial of Autologous Blood products, leukocyte and platelet-rich fibrin (L-PRF), to promote ulcer healing in LEprosy: The TABLE trial
Source: PLoS Negl Trop Dis. 2024 May 2;18(5):e0012088. doi: 10.1371/journal.pntd.0012088 (PMC11093377; doi:10.1371/journal.pntd.0012088)

**S2 Figure.**  Bland-Altman plots assessing inter-observer agreement for the three measurement methods


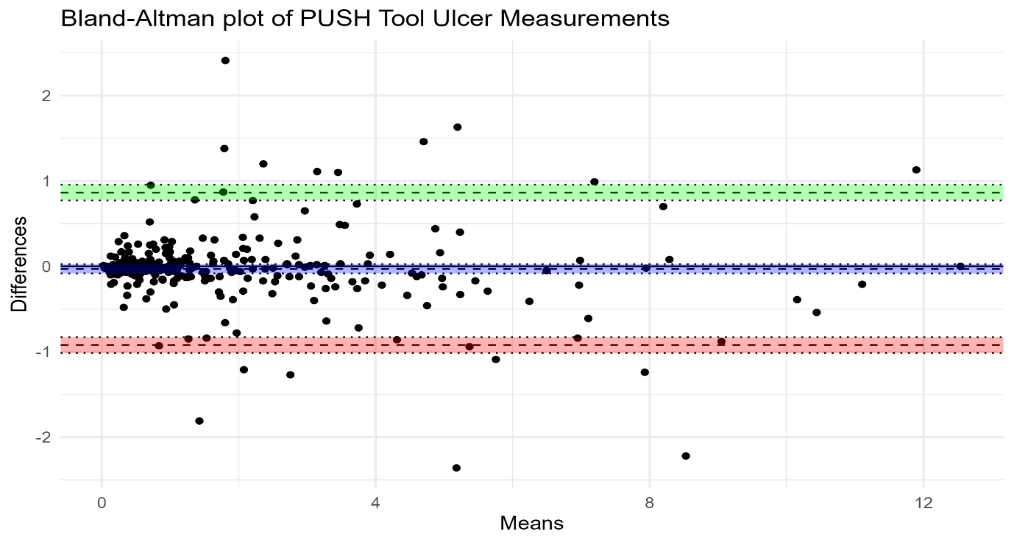


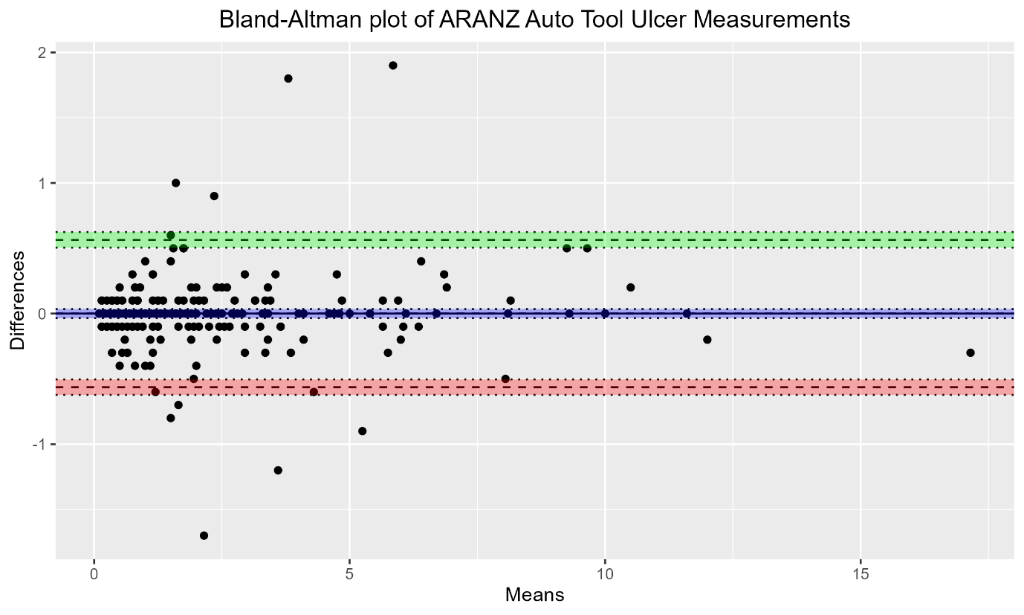


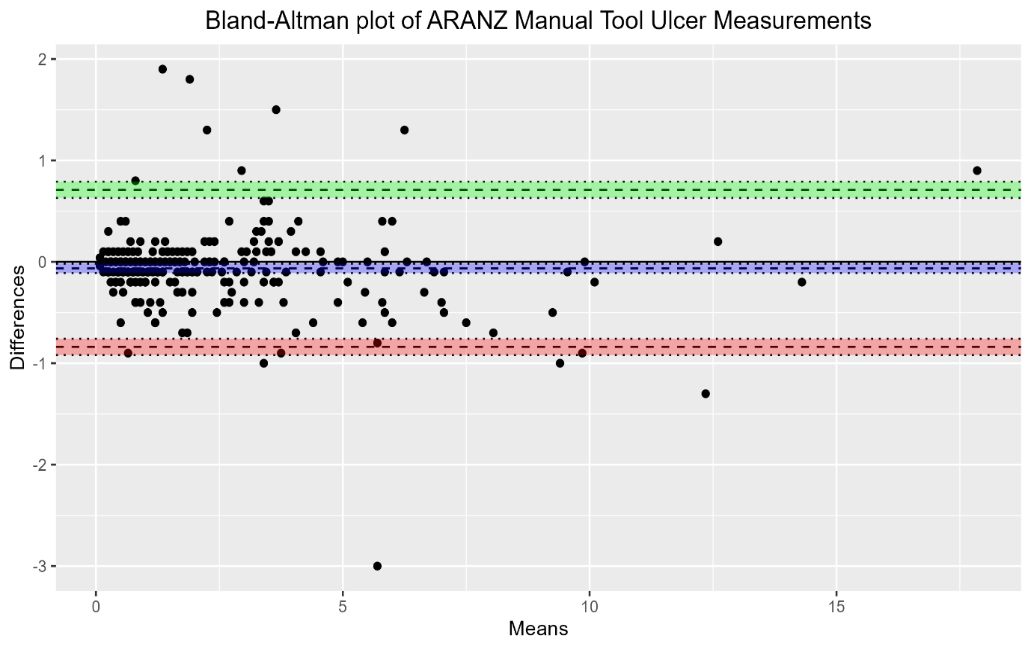

Supplement: S2 Fig — (DOCX) [file pntd.0012088.s020.docx]
