# Supplementary material for: A randomised Trial of Autologous Blood products, leukocyte and platelet-rich fibrin (L-PRF), to promote ulcer healing in LEprosy: The TABLE trial
Source: PLoS Negl Trop Dis. 2024 May 2;18(5):e0012088. doi: 10.1371/journal.pntd.0012088 (PMC11093377; doi:10.1371/journal.pntd.0012088)
Supplement: Attachment — Submitted filename: Response to comments on email from 14.02.2024.docx [file pntd.0012088.s021.docx]

Dear Reviewers,

Thank you very much for your comments on TABLE trial manuscript, manuscript ID: PNTD-D-23-01402R1. Please, find the justifications for the comments below. Thanks.

Dr Indra

| Comments | Justification |
| --- | --- |
| 1) We ask that a manuscript source file is provided at Revision. Please upload your manuscript file as a .doc, .docx, .rtf or .tex. If you are providing a .tex file, please upload it under the item type ‘LaTeX Source File’ and leave your .pdf version as the item type ‘Manuscript’.  2) Tables should not be uploaded as individual files. Please remove these files and include the Tables in your manuscript file as editable, cell-based objects. For more information about how to format tables, see our guidelines: <https://journals.plos.org/plosntds/s/tables>  3) We notice that your supplementary materials (Appendix 10,Appendix 13)  are uploaded with the file type 'Figure'. Please amend the file type to 'Supporting Information'. Please ensure that each Supporting Information file has a legend listed in the manuscript after the references list.  4) "Please upload all main figures as separate Figure files in .tif or .eps format. For more information about how to convert and format your figure files please see our guidelines: <https://journals.plos.org/plosntds/s/figures>  5) Please ensure that the CRediT author contributions listed for every co-author are completed accurately and in full. At this stage, "Several Authors" require contributions. Please ensure that the full contributions of each author are acknowledged in the ""Add/Edit/Remove Authors"" section of our submission form.  The list of CRediT author contributions may be found here:  <https://journals.plos.org/plosntds/s/authorship#loc-author-contributions>  6) In the online submission form, you indicated that [The full protocol, non-identifiable participant level data and statistical code will be available on request. ].   All PLOS journals now require all data underlying the findings described in their manuscript to be freely available to other researchers, either 1. In a public repository, 2. Within the manuscript itself, or 3. Uploaded as supplementary information.  This policy applies to all data except where public deposition would breach compliance with the protocol approved by your research ethics board. If your data cannot be made publicly available for ethical or legal reasons (e.g., public availability would compromise patient privacy), please explain your reasons by return email and your exemption request will be escalated to the editor for approval. Your exemption request will be handled independently and will not hold up the peer review process, but will need to be resolved should your manuscript be accepted for publication. One of the Editorial team will then be in touch if there are any issues.  7) Thank you for stating" All requests will be approved by the Chief Investigator (CI), Professor Richard Lilford ([r.j.lilford@bham.ac.uk](mailto:r.j.lilford@bham.ac.uk))".  In the online submission form you indicate that your data is not available for proprietary reasons and have provided a contact point for accessing this data. Please note that your current contact point is a co-author on this manuscript. According to our Data Policy, the contact point must not be an author on the manuscript and must be an institutional contact, ideally not an individual. Please revise your data statement to a non-author institutional point of contact, such as a data access or ethics committee, and send this to us via return email. Please also include contact information for the third party organization, and please include the full citation of where the data can be found.  8) "Please provide an Author Summary. This should appear in your manuscript between the Abstract (if applicable) and the Introduction, and should be 150–200 words long. The aim should be to make your findings accessible to a wide audience that includes both scientists and non-scientists. Sample summaries can be found on our website under Submission Guidelines: <https://journals.plos.org/plosntds/s/submission-guidelines#loc-parts-of-a-submission>  9) We do not publish any copyright or trademark symbols that usually accompany proprietary names, eg (R), (C), or TM  (e.g. next to drug or reagent names). Please remove all instances of trademark/copyright symbols throughout the text, including PictZar™ on line no 234, ARANZ® on line no 218, 219,220,224.  10) We have noticed that you have uploaded Supporting Information files, but you have not included a list of legends. Please add a full list of legends for your Supporting Information files after the references list.  11) Thank you for stating" A patient information leaflet was provided and the potential participant was invited to sign a consent form on the following day".  Please be so kind and specifically state whether written consent (sign a consent form) was obtained from the patients?  12) Please amend your detailed Financial Disclosure statement. This is published with the article, therefore should be completed in full sentences and contain the exact wording you wish to be published. i). State what role the funders took in the study. If the funders had no role in your study, please state: “The funders had no role in study design, data collection and analysis, decision to publish, or preparation of the manuscript.” ii). If any authors received a salary from any of your funders, please state which authors and which funders. Once you have made the necessary changes in Editorial Manager, please re-approve the submission by following the same steps as before. | Manuscript uploaded as a .doc file.  The main Tables in the manuscript are added as editable, cell-based objects. Other tables are uploaded as supplementary information. A legend list added in the manuscript after the references list. Page 32, line 724.  Supplementary materials (Appendix 10, Appendix 13) with file type figure uploaded as supporting information figures. A legend list added in the manuscript after the references list.  All main figures uploaded as .tif files.  Author contribution listed for every co-author.  The data underlying the findings would require participant level trial data being made publicly available which could compromise patient privacy, and participants did not consent for their data to be made publicly available.  We are however able to share non-identifiable participant level data upon request with an appropriate data sharing agreement in place.  [bctudatashare@contacts.bham.ac.uk](mailto:bctudatashare@contacts.bham.ac.uk)  For more information, please click at the link: <https://www.birmingham.ac.uk/research/bctu/data-sharing-and-protection-policy>  Added Author Summary at page 3**.**  Removed all instances of trademark/copyright symbols.  A legend list added in the manuscript after the references list.  Yes, written consent was obtained from the patients. Added in the manuscript. Page 6, line 115.  The funders had no role in study design, data collection and analysis, decision to publish, or preparation of the manuscript.  All authors received salary from the funder- NIHR, UK. |
